# Cullin3 deficiency shapes tumor microenvironment and promotes cholangiocarcinoma in liver-specific Smad4/Pten mutant mice

**DOI:** 10.7150/ijbs.67379

**Published:** 2021-10-11

**Authors:** Ming Zhao, Yingyao Quan, Jianming Zeng, Xueying Lyu, Haitao Wang, Josh Haipeng Lei, Yangyang Feng, Jun Xu, Qiang Chen, Heng Sun, Xiaoling Xu, Ligong Lu, Chu-Xia Deng

**Affiliations:** 1 Cancer Center, Faculty of Health Sciences, University of Macau, Macau SAR, China.; 2 Centre for Precision Medicine Research and Training, Faculty of Health Sciences, University of Macau, Macau SAR, China.; 3 Department of Oncology, The Affiliated Hospital of Southwest Medical University, Luzhou, China.; 4 Zhuhai Interventional Medical Center, Zhuhai Precision Medical Center, Zhuhai People's Hospital, Zhuhai Hospital Affiliated with Jinan University.; 5 MOE Frontieers Science Center for Precision Oncogene, University of Macau, Macau SAR, China.

**Keywords:** Cholangiocarcinoma, tumor microenvironment, inflammatory cytokines, exhausted T cells, anti-PD1/PD-L1 therapy

## Abstract

Cholangiocarcinoma (CC), the most lethal type of liver cancer, remains very difficult to treat due to an incomplete understanding of the cancer initiation and progression mechanisms and no effective therapeutic drugs. Thus, identification of genomic drivers and delineation of the underlying mechanisms are urgently needed. Here, we conducted a genome-wide CRISPR-Cas9 screening in liver-specific Smad4/Pten knockout mice (*Smad4^co/co^;Pten^co/co^;Alb-Cre,* abbreviated as SPC), and identified 15 putative tumor suppressor genes, including Cullin3 (Cul3), whose deficiency increases protein levels of Nrf2 and Cyclin D1 that accelerate cholangiocytes expansion leading to the initiation of CC. Meanwhile, Cul3 deficiency also increases the secretion of Cxcl9 in stromal cells to attract T cells infiltration, and increases the production of Amphiregulin (Areg) mediated by Nrf2, which paracrinely induces inflammation in the liver, and promotes accumulation of exhausted PD1^high^ CD8 T cells at the expenses of their cytotoxic activity, allowing CC progression. We demonstrate that the anti-PD1/PD-L1 blockade inhibits CC growth, and the effect is enhanced by combining with sorafenib selected from organoid mediated drug sensitive test. This model makes it possible to further identify more liver cancer suppressors, study molecular mechanisms, and develop effective therapeutic strategies.

## Introduction

Cholangiocarcinoma (CC), a malignant adenocarcinoma, is the second most common primary hepatic malignancy with bile duct epithelial differentiation 1, 2. It accounts for approximately 15% of the total liver cancer cases, and both the incidence and mortality have continued to increase worldwide in recent decades 3, 4. The prognosis of CC is poorer than that of hepatocellular carcinoma (HCC), with a median survival rate of less than 1 year after diagnosis 5. Despite extensive studies that have been conducted to study the CC initiation, progression and drug responses 6, 7, the mechanisms underlying poor survival and refractory nature of CC remain elusive, which hinders the development of effective therapies.

A number of genetic mutations have been identified in human CC specimens, such as the tumor suppressors P53, P16, Smad4 and Pten as well as oncogenes Ras, Ctnnb1, Cyclin D1, Akt and c-Myc 8, 9. However, in a genomic profiling of 200 human CC specimens, majority of them (61.5%) did not show any known mutations 10, suggesting that many more unknown mutations might be involved in tumor initiation and progression and highlighting the need to perform a large-scale screen of the unknown genetic mutations driving tumorigenesis.

Transposon and lentivirus insertional mutagenesis have been the main methods for *in vivo* genetic screening in the last decade 11, 12. However, although they both could cause loss- or gain-of-function effects to identify tumor suppressors or oncogenes, they are not widely used because of insertional biases and low efficiency. Recently, the pooled genome-wide scale CRISPR knockout library has been shown to be a robust strategy to screen for tumor suppressors or metastatic genes *in vitro* or in xenograft mouse models 13-16. However, this system has not been applied for *in vivo* genetic screening for liver tumor suppressors.

Smad4 is a central mediator of the TGF-β signaling pathway and plays a pivotal role in cancer initiation and progression 17. We previously conducted liver-specific knockout of Smad4 using a Cre-LoxP system, which demonstrates that the loss of Smad4 alone failed to induce liver cancer formation, although it caused iron accumulation in many organs/tissues as a consequence of markedly reduced hepcidin production in the liver 18. On the other hand, liver-specific deletion of Pten, which is a well-known tumor suppressor in a wide range of human cancers 19, also failed to induce liver cancer formation in one year, although it caused severe steatosis during the early stages. We found that after liver-specific knockout of both Smad4 and Pten using the Cre-LoxP mediated approach, *Smad4^co/co^;Pten^co/co^;Alb-Cre* (abbreviated as SPC) mice started to develop hyperplasia at 2-3 months old, and approximately 22% (2/9) of SPC mice developed CC at 5-6 months old, with some mice also developing HCC after one year of age 7. This observation suggests that even with both Smad4 and Pten deficiencies, liver cancer still appears in a stochastic fashion, perhaps pending further alterations of additional drivers.

To identify the additional drivers in liver cancer formation, we conducte CRISPR-Cas9-mediated genome-wide screening in SPC mice. We identify a set of putative tumor suppressor genes and demonstrate that Cul3 is a suppressor in CC tumorigenesis by inducing expression of oncogenes, as well as promoting exhausted PD1^high^ CD8 T cells accumulation in the hepatic microenvironment, creating a favorable tumor microenvironment that facilitates CC initiation and progression. The anti-PD1/PD-L1 antibodies combined with sorafenib treatment represents an effective therapeutic strategy.

## Materials and methods

### Vectors

The following vectors were obtained from Addgene: pCDH-EF1-Luc2-P2A-copGFP (GFP-Luc, #72485), PX330 (#42230), lenti-CRISPR v2 (#52961) and the mouse genome-scale CRISPR knockout library (#1000000052). The sgRNAs targeting individual genes that used in candidate validation were cloned by inserting the specific oligos of the target sequence into PX330 according to the standard protocol 20.

### Lentivirus production and concentration

The lentivirus was produced by transfecting GFP-Luc or CRISPR knockout library plasmids (10 µg) into HEK293FT cells in a 15-cm dish together with the viral packing plasmid Delta8.2 (7.5 µg) and the envelope plasmid VSVG (5 µg). The supernatant containing viral particles was harvested two times at 48 hours and 60 hours. All the supernatants were filtered through 0.45 μm polyethersulfone (PES) filters and pooled together to be concentrated at 70,000 g for 1.5 hours at 4 °C by Optima XPN-90 Ultracentrifuge (Beckman). The pellet was washed once with PBS, and the virus was resuspend again in PBS. The suspension was divided into aliquots, which were stored at -80 °C for less than 1 month.

### Animal experiments

All mouse experiments are approved by the Animal Care and Use Committee of University of Macau (UMAEC-050-2015). Lentivirus (1 × 10^9^ in 200 µl PBS) was injected into 3-4-week-old SPC mice by tail vein injection. For single sgRNA delivery, the constructed vector PX330 (30 μg) was dissolved in 1.8 ml sterile PBS and rapidly injected into four-week-old mice via the tail vein within 5-8 seconds as reported 21, 22. For tumor number counting, some tumors grew as clusters and they were hard to count; thus, the tumor number was considered to be at least 20. For the drug treatment *in vivo*, sorafenib was administerd orally every other day starting from age of 2 month, and anti-PD1 antibody by ip. injection every week starting from age of 1 month.

### Cell culture

The CC cell line (273cc) was isolated from SPC mice. The doxycycline (abbreviated as Dox)-inducible shRNA plasmid was constructed and packaged to be lentivirus, which was followed by infecting 273cc. Dox was added at 1 µg/ml, and the mRNA or protein level was detected in the following days.

### Histology and immunohistochemistry

Livers or tumors were harvested and fixed in 10% (v/v) formalin overnight and embedded in paraffin. Antigen retrieval was performed in R-BUFFER A (Electron Microscopy Sciences) using a vegetable streamer. The following antibodies were used: AE-1 (1:200, Signet Laboratories), Hep Par1 (1:100, Dako), AFP (1:200, Abcam), GFP (1:200, Thermos Fisher Scientific), PCNA (1:10000, Cell Signaling Technology), and BrdU (1:200, Santa Cruz Biotechnology).

### Western blot analysis

Total protein lysates from liver or tumor tissues were extracted by RIPA lysis buffer supplemented with proteinase inhibitor and phosphatase inhibitor (Roche Diagnostics). The samples were separated on a 10% SDS-PAGE gel and then transferred to a nitrocellulose membrane (Millipore), followed by probing with the following antibodies: Cul3 (1:1000, Bethyl Laboratories), Cyclin D1 (1:1000, Cell Signaling Technology), Stat1, p-Stat1 (1:1000, Cell Signaling Technology), PCNA (1:10000, Cell Signaling Technology) and GAPDH (1:2000, Santa Cruz Biotechnology).

### RNA purification and quantitative real-time PCR

Total RNA was extracted from liver or tumor tissues using TRIzol (Invitrogen) according to the provided protocol. First-strand cDNA was synthesized using the QuantiTect Reverse Transcription Kit (Qiagen) and as the template to be amplified using FastStart Universal SYBR Green Master (Rox) (Roche Diagnostics). The mRNA levels of the target genes were normalized to that of 18S rRNA.

### Flow cytometry

To obtain single cell suspensions, the livers were minced and incubated with DMEM containing 2 mg/ml collagenase IV and 1.2 mg/ml of collagenase II. After one hour of digestion, the cells were washed with HBSS and stained with surface-staining antibodies, including anti-CD45 APC, anti-CD3e FITC, anti-CD8a APC, anti-CD279 PE. For anti-IFNγ PE staining, cells were pretreated with Fix/Perm buffer (eBioscience, San Diego, CA) for 30 min at room temperature.

### CyTOF analysis of livers

Single-cell suspensions (2 × 10^7^ cells/ml) in serum-free medium were incubated with an equal volume of 10 µM cisplatin for 5 min at room temperature. After washing with Maxpar Cell Staining Buffer, 3 million cells were resuspended in 80 µl Maxpar Cell Staining Buffer and incubated with 20 µl Fc-receptor blocking solution for 10 min at 4 °C. Then, the cells were washed again and incubated with the mixed antibody cocktail in 100 µl Maxpar Cell Staining Buffer for 30 min at room temperature, followed by CELL-ID Intercalator-Ir in Maxpar Fix and Perm Buffer overnight at 4 °C. Before running on the Helios mass cytometer (Fluidigm), the cells were washed with deionized water and mixed with EQ Four Element Calibration Beads labeled with 140Ce. All the antibodies are from DVS-Fluidim: Ly6G-141Pr, CD11c-142Nd, CD69-143Nd, CD4-145Nd, CD8a-146Nd, CD45-147Sm, CD11b-148Nd, CD19-149Sm, CD3e-152Sm, CD14_156Gd, Foxp3-158Gd, F4_80-159Tb, CD62L-160Gd, Ly_6C-162Dy, Arg1-164Dy, EpCAM-165Ho, CD206-169Tm, NK1.1-170Er, CD44-171Yb, CD86-172Yb, IA_IE-174Yb, CD3e-152Sm, CD4-145Nd, CD8a-146Nd, Ly-6G-141Pr, Ly6C-162Dy, CD45-147Sm, CD11b-148 Nd, CD279-159Tb, CD274-153Eu, Ifnγ-165Ho, CD28-151Eu. CyTOF data analysis was gated by non-bead and live/dead discrimination in FlowJo. The exported files were imported into R for downstream analysis.

### Sequence of sgRNA enrichment

Q5 Hot Start High-Fidelity (NEB) was used to amplify the DNA fragments with 300 bp covering the guide sequence, and the fragments were purified by a Precellys Ceramic Kit (Peqlab). Different paired barcodes were ligated to the fragment by a secondary round PCR in each tumor. All the barcoded samples were pooled to be sequenced. The sequenced raw data were demultiplexed and trimmed to be 20 nt sgRNA sequences, followed by mapping with sgRNA sequences in the CRISPR knockout library. MAGeCK 23 was used to identify positively and negatively selected sgRNAs and genes in genome-scale CRISPR/Cas9 knockout experiments. The method consists of read count normalization, sgRNA ranking and gene ranking, and the enriched sgRNAs are shown in a ranked list.

### scRNA-Seq library construction using the 10× Genomics platform

The scRNA-Seq library was prepared with Single Cell 3' Reagent Kits v2 (10× Genomics, PN-120237), following the standard photometric methods in the kit. Briefly, 8,000 single cells were encapsulated into droplets, followed by lysis and reverse transcription in a Thermal cycler. Then, the barcoded-cDNA was released by broking the droplets and purified with DynaBeads (Thermo Fisher Scientific; 37002D). The cDNA was amplified by PCR with 13 cycles, and then fragmented and ligated with adaptor and sample index. At last, fragments at about 300bp were selected with SPRI beads (Beckman Coulter; B23318).

### scRNA-Seq analysis

Cell Ranger pipeline (v4.0) introduced by the 10X Genomics was firstly employed to process single cell RNA sequencing data with default parameters. Raw read count matrixes were integrated and normalized by Seurat (v3.2.2). Next high-variance genes identified by FindVariableGenes were chosen and dimensional reduction was applied for downstream analysis. Then, cells were clustered together according to their gene expression level. Specific markers were used to identify cell clusters. AUCell package (v1.12.0) was used to calculate gene expression signature score.

### Tumor organoid culture

The isolated single cells were washed in cold advanced DMEM/F12 and were seeded in Matrigel. Thirty minutes after incubation at 37 °C, the Matrigel was solidified, and growth medium was added. The composition of the medium was advanced DMEM/F-12 supplemented with 1% penicillin/streptomycin, 1% GlutaMAX, 10 mM HEPES, 2% B-27, 1% N-2, 10 mM nicotinamide, 1.25 mM N-acetyl-L-cysteine, 10 nM [Leu15]-gastrin, 10 mM forskolin, 5 mM A83-01, 50 ng/mL EGF, 25 ng/ml HGF, 0.1% Plasmocin, 10% RSpo1-conditioned medium (homemade), and 30% Wnt3a-conditioned medium (homemade). The culture medium was changed twice a week.

### Drug screening with the organoids

Tumor organoids were dissociated with 0.25% trypsin-EDTA and mechanically pipetted up and down before being resuspended in growth media (15,000-20,000 cells/ml). Briefly, 12 μl of 1.3 mg/ml collagen I was dispensed into each well of 384-well microplates. Ten minutes after incubation at 37 °C for polymerization, the dissociated organoids were dispensed into 384-well plates. Twenty-four hours later, each compound at a series concentration was dispensed, and cell viability was assayed using an ATP Assay Kit (Abcam) after 96 hours of incubation. The maximum concentration of each compound was 20 µM, and the experimental concentration range was calculated using a three-point half-log dilution series of the highest maximum concentration.

### Statistics

GraphPad Prism 7 was used for statistical analysis. Samples were analyzed by using a paired or unpaired two-tailed t test. All values are expressed as the mean ± SD. Kaplan-Meier survival data were analyzed by a log-rank (Mantel-Cox) test.

## Results

### Lentiviral delivery of the CRISPR knockout library markedly accelerates CC in SPC mice

**Figure 1:**
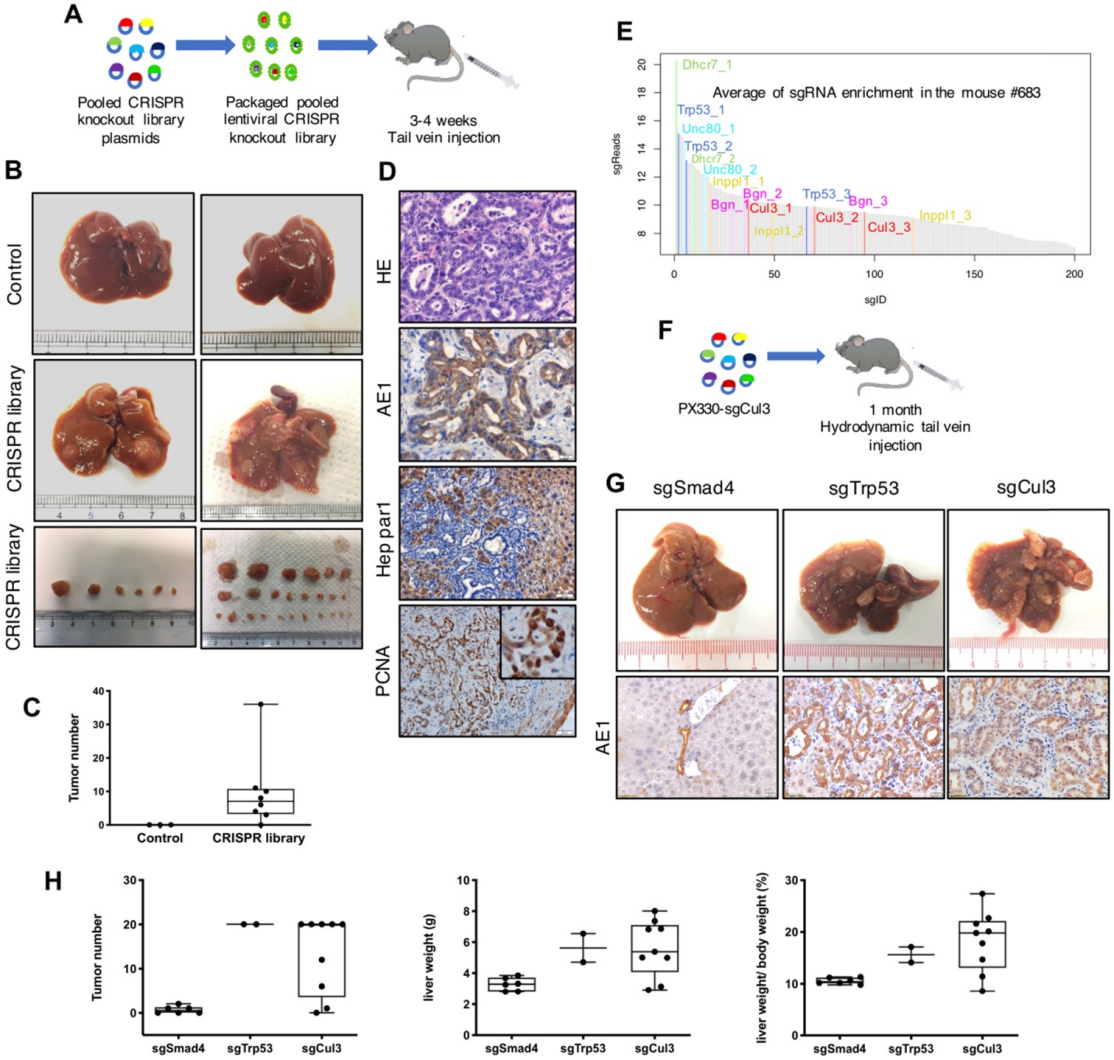
** Lentiviral delivery of the CRISPR knockout library identifies Cul3 as a suppressor of CC in SPC mice. (A)** Outline of tail vein injection of lentivirus-mediated CRISPR knockout library. **(B)** Livers injected with the CRISPR knockout library develop tumors at 3-4 months, while the control livers are normal. **(C)** Summary of tumor numbers in each mouse. **(D)** Histology analysis of tumors induced by the CRISPR knockout library. AE1 indicates cholangiocytes and Hep Par1 indicates hepatocytes. PCNA represents cell proliferation. **(E)** Plot shows the average of sgRNA enrichment measured by high-throughput sequencing. **(F)** Outline of hydrodynamic tail vein injection of sgRNA. **(G)** Up panel: Representative images of the livers injected with sgRNAs targeting Smad4, Trp53 and Cul3. Down panel: Immunostaining of paraffin sections show the phenotype of CC. **(H)** Statistics of the tumor number, liver weight and the ratio of the liver weight to body weight (LW/BW).

To check the infection efficiency of the lentivirus in the mouse liver, we injected concentrated GFP-Luc lentivirus by tail vein, and it showed an obvious signal during the following week (Fig. S1A-C). Next we injected the lentivirus carrying CRISPR knockout library into 22-28 day (3.5 week) old SPC mice through the tail vein (Fig. 1A), and 8 out of 9 recipient mice developed visible liver tumors at autopsy at 3-4 months of age, with tumor numbers ranging from 2 to 36 per mouse (Fig. 1B, C). Histological analysis indicated that all tumors were CC (Fig. 1D). Lentivirus with nontargeting sgRNA were utilized as a parallel control to exclude the possibility that lentivirus itself could induce tumor formation (n=3) (Fig. 1B, C). These results revealed that tail vein injection of lentivirus carrying the CRISPR knockout library markedly accelerates CC formation in SPC mice.

### Identification of Cul3 as a suppressor of CC in SPC mice

To identify genes that are responsible for accelerated CC formation, we evaluated sgRNA enrichment in tumors by next-generation sequencing (NGS). Because each gene in the library was targeted by 3 sgRNAs, we selected genes with 2 or 3 corresponding sgRNAs in top 200 sgRNA enrichment list. With this criteria, we identified sgRNAs for 15 genes (Fig. 1E, Table 1). Among these genes, deletion of Trp53 had already been reported to be able to induce CC formation in Pten-deficient mice 24, 25, while mutations of the remaining genes had not yet been reported.

For validation, plasmids carrying sgRNA for individual genes were delivered by hydrodynamic tail vein injection (Fig. 1F), which is a reliable method to deliver plasmids to the liver (Fig. S1D-F). We included sgTrp53 as a positive control and sgSmad4 as a negative control, which had already been knocked out in the recipient SPC mice. As a result, 4 out of 9 genes tested could induce tumor formation, including sgCul3, which exhibited compariable ability in inducing CC formation with sgTrp53 (Fig. 1G, H). Notably, the tumors sometimes appeared as early as 1 month after sgCul3 injection, and additional tumors grew out in the next 1 or 2 months (Fig. S2A-D). To evaluate the sgCul3-targeting site, tumor were isolated to get DNA for NGS, showing indels that would lead to frameshift and inactivation of Cul3 (Fig. S2E). Additionally, Western blot results showed lower Cul3 protein level in the tumors of SPC;sgCul3 mice at 3-4 months than in the tumors of SPC mice at 5-6 months (Fig. S2F).

To provide further evidence for Cul3 in suppressing CC formation, we injected a Cre expression plasmid and the sgCul3 construct to *Smad4^co/co^;Pten^co/co^* (SP) mice (Fig. S3A). The data indicated that 3 months after the injection, the SP mice developed liver tumors, while the mice injected with only Cre were normal (Fig. S3B, C). The deletion of Smad4 and Pten by the Cre plasmid in the tumor was confirmed by PCR using specific primers (Fig. S3D, E). The tumor was evaluated by NGS, and the DNA showed variation in the indels and percentage of each read (Fig. S3F). The histological analysis showed the subtype of CC (Fig. S3G).

### Cul3 deficiency accelerates cholangiocyte expansion at an early stage

**Figure 2:**
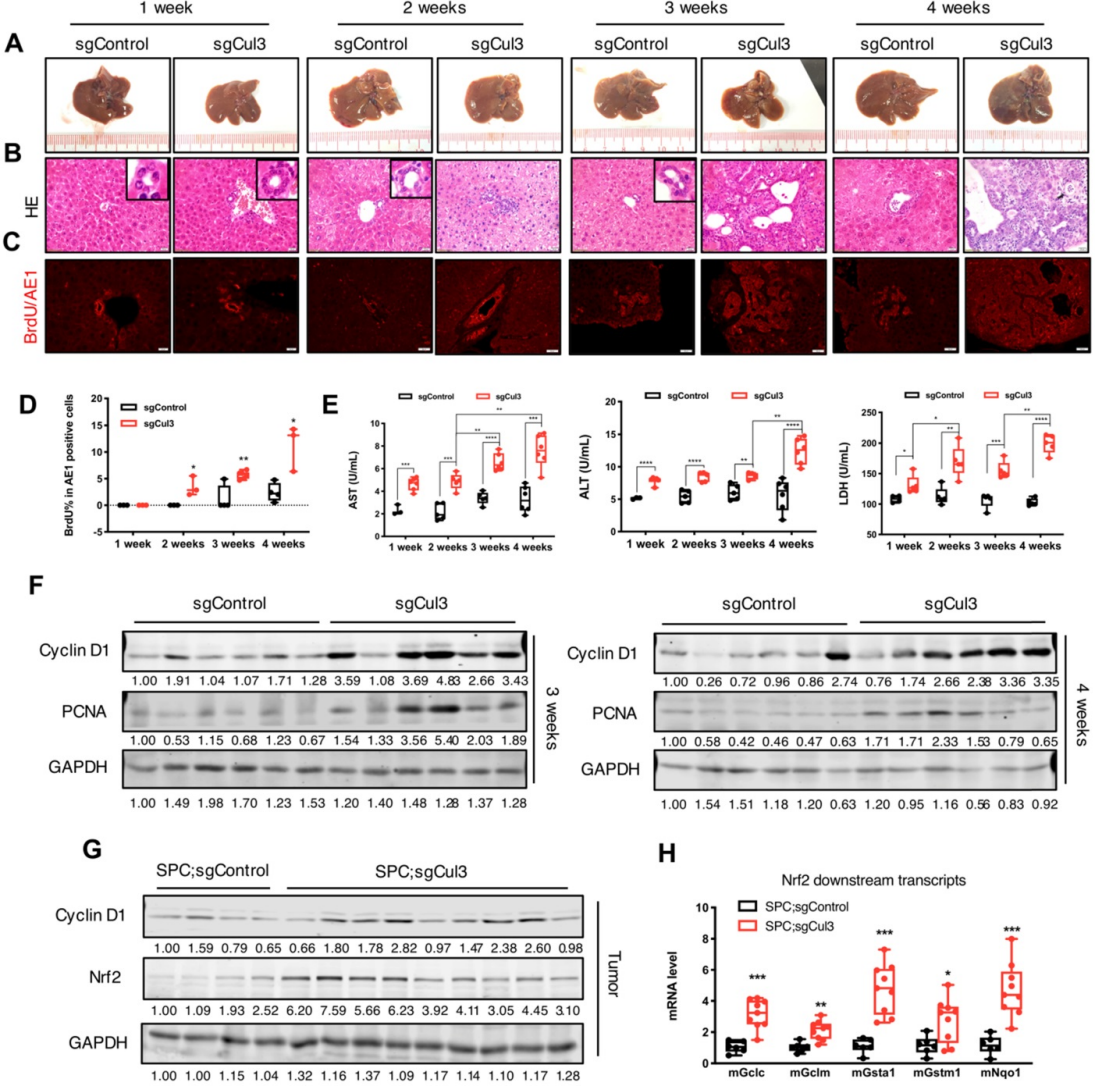
** Cul3 deficiency accelerates cholangiocyte cell expansion at an early stage. (A)** Macroscopic images of livers injected with sgControl or sgCul3. **(B)** H&E staining of paraffin sections show the morphology of bile duct expansion. **(C)** Co-staining of AE1 and BrdU incorporation. AE1 is stained with a red signal on the surface, while BrdU is shown as a red dot in the nucleus. **(D)** Quantification of BrdU incorporation in AE1-positive cholangiocytes (n≥3 at each time point). **(E)** Analysis of biochemical markers of liver injury released in blood (n≥3 at each time point). **(F)** Western blot analysis results show increased protein levels of pro-tumorigenic molecules. **(G)** Western blot shows higher Nrf2 protein levels in tumors from SPC;sgCul3 mice at 3-4 months compared with tumors from SPC;sgControl mice at 6-7 months. **(H)** qRT-PCR shows activated Nrf2 downstream transcripts (n=5 for SPC;sgControl mice, n=9 for SPC;sgCul3 mice). *P<.05, **P<.01, ***P<.001, ****P<.0001.

To identify the earliest time point when the disruption of Cul3 accelerates tumorigenesis, we examined the liver at 1, 2, 3 and 4 weeks after an injection of sgCul3. Until at least the first three weeks after injection, the livers did not show any observed nodules on the surface (Fig. 2A). However, H&E and AE1 staining of sections at 2 weeks sometimes revealed disorganized bile ducts and enhanced proliferation in sgCul3 injected livers, as indicated by increased BrdU incorporation (Fig. 2B-D).

We next evaluated the biochemical molecules of liver injury that were released into the blood (Fig. 2E). Strikingly, all three markers, including alanine transaminase (ALT), aspartate aminotransferase (AST), and lactate dehydrogenase (LDH), were higher at 1 week in the sgCul3 mice than in the sgControl mice, and continued to increase over the following weeks.

To study the underlying mechanism for accelerated tumorigenesis upon Cul3 disruption, we examined expression of the pro-tumorigenic gene Cyclin D1 and the proliferation marker PCNA, both of which were significantly upregulated in the sgCul3 livers compared with that in sgControl livers at 3 and 4 weeks after the injection (Fig. 2F). We also examined expression of Nrf2, which is a well-known target of Cul3 26 and is known to be involved in several types of cancers, including HCC 27. We found the upregulation of Nrf2 and its downstream genes, consistent with the biological influence of Cul3 loss (Fig. 2G, H). Altogether, these results suggest that the inactivation of Cul3 accelerates cholangiocyte expansion at an early stage through affecting some of its downstream oncogenic signaling.

### Cul3 deficiency induces inflammation in the liver

**Figure 3:**
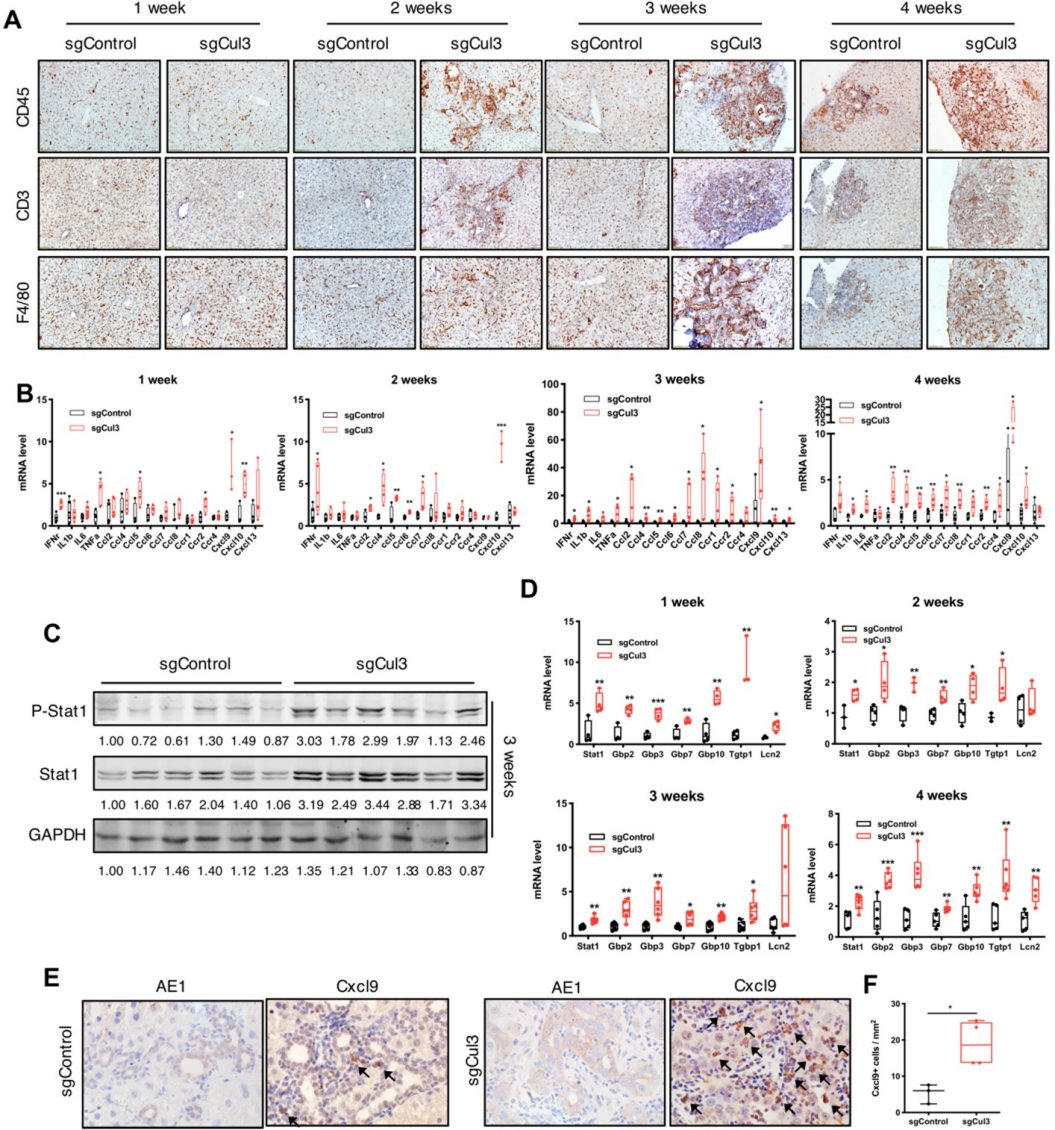
** Cul3 deficiency induces inflammation in the liver. (A)** Representative immunohistochemistry of immune cells in livers. CD45 indicates lymphocytes; CD3 indicates T cells; F4/80 indicates macrophages. **(B)** qRT-PCR shows mRNA levels of inflammatory cytokine genes. **(C)** Western blot and **(D)** qRT-PCR results reveal the activated Stat1 pathway. **(E)** Representative immunostaining of Cxcl9 and AE1 in foci of the liver at 4 weeks after injection with sgControl or sgCul3, and the Cxcl9-positive cells are quantified in **(F)**. Arrow indicates Cxcl9 signal. *P<.05, **P<.01, ***P<.001, ****P<.0001.

We next examined the dynamic changes in the tumor microenvironment during the first 4 weeks after the injection of sgCul3. The distribution of immune cells, including T cells and macrophages, showed more infiltration into the cholangiocyte area and continuous expansion at 2 weeks after the sgCul3 injection (Fig. 3A). We next examined the inflammatory cytokines by qRT-PCR and observed upregulation at 1 week after sgCul3 administration. Almost all cytokines were upregulated after 2 weeks at various levels, most significantly Ccl2, -4, -5, -6, -7, and -8 and Cxcl9 and -10 (Fig. 3B).

Of note, our Western blot analysis of livers at early time points after sgCul3 administration revealed increased levels of Stat1 and its phosphorylation (Fig. 3C), which is known to play a role in promoting inflammation 28, 29. qRT-PCR analysis also detected significantly increased transcripts of Stat1 and its downstream genes (Fig. 3D), which is corresponding to the enhanced inflammation in SPC;sgCul3 mice. Cxcl9, a downstream gene of the Stat1 pathway 30, has been reported to be a key chemokine for cytotoxic T cell infiltration 31, 32. To investigate its origin in the tumor area, we stained Cxcl9 in foci of the liver at 4 weeks after injection of sgCul3 and demonstrated its expression in stromal cells (Fig. 3E). The data further confirmed the higher density of the Cxcl9 signal in the foci of SPC;sgCul3 mice than in the foci of SPC;sgControl mice (Fig. 3E, F).

To investigate whether the inflammation phenomenon is also associated with tumor progression, we conducted transcriptome analyses using tumors isolated from 3-month old SPC;sgCul3 mice and 6-month-old SPC mice (for comparing tumors at approximately equal size) (Fig. S4A). It revealed extensive alterations of inflammation-related pathways, including the interferon γ and interferon α response pathways (Fig. S4B, C), and the inflammatory cytokine upregulation, including ligand-receptor pairs, such as Ccl5-Ccr5 and Cxcl9/Cxcl10-Cxcr3 (Fig. S4D). Further analysis also showed the upregulation of Stat1 and its phosphorylation (Fig. S4E, F). These data provide compelling evidence that key alterations triggered by Cul3 deficiency not only at the early stages of CC initiation, but also is associated with its progression.

### scRNA-Seq reveals reduced activity of cytotoxic T cells in tumors from SPC;sgCul3 mice compared with that in SPC;sgControl mice

To investigate the immune microenvironment in tumors from both SPC;sgControl and SPC;sgCul3 mice, we digested four tumors in each group and performed scRNA-seq using the 10× Genomics platform (Fig. S5A). After dimension reduction and iterative clustering, we got eight major cell clusters, including T cell, B cell, macrophage, neutrophil, dendritic cell, endothelial cell, epithelial cell and fibroblast (Fig. S5B, C). In the tumors, macrophage and neutrophil are the first two major cell types, however, without significant change of percentage. Surprisingly, the T cells, which are the main cytotoxic cells against tumors, showed about 2-fold higher in SPC;sgCul3 mice than that in SPC;sgControl mice (Fig. S5D, E).

**Figure 4:**
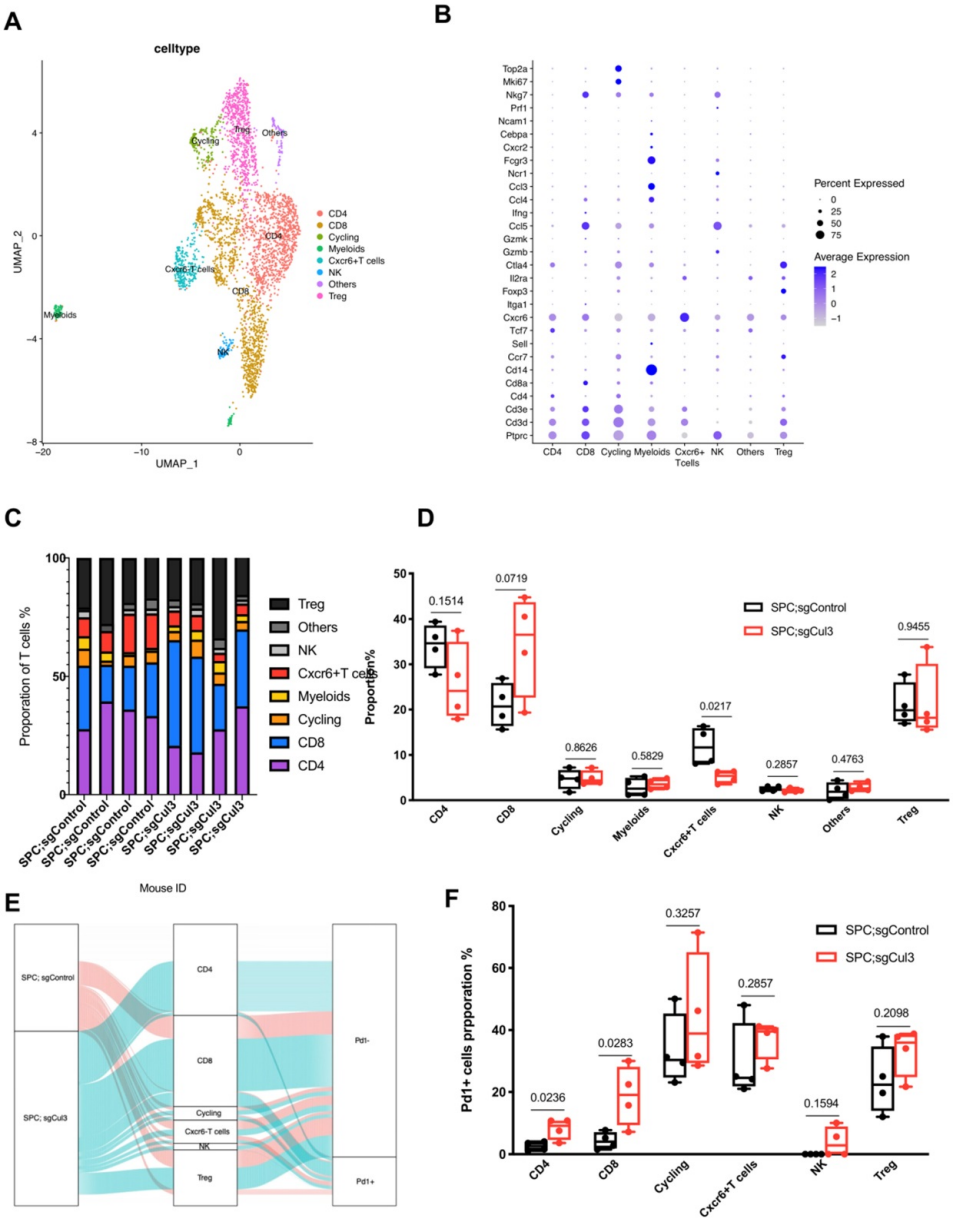
** Increased accumulation of Pd1+ CD8 T cells in tumors from SPC;sgCul3 mice compared with that in SPC mice. (A)** t-SNE plots showing the identified cell clusters. **(B)** Dot plot showing the expression levels of marker genes for each cell population. **(C)** Cell cluster frequency for each mouse. **(D)** Comparison of cell lineages in each group. **(E)** Sankey diagram showing the Pd1 positive or negative fraction in each cluster from SPC and SPC;sgCul3 mice. **(F)** The frequency of Pd1+ T cells in SPC and SPC;sgCul3 mice (n=4).

We next extracted CD3 T cells from the single cell data, and subclustered into groups including CD8 T cell, CD4 T cell, Treg, NK, Cxcr6+ T cell, cycling, myeloids and CD3 negative cells (others) (Fig. 4A, B). The percentage of CD8 T cell increased to about 1.5-fold higher in SPC;sgCul3 mice than SPC mice, while the Cxcr6+ T cell decreased by half (Fig. 4C, D). To further investigate the phenotype of T cell subtypes, we analyzed the fraction of Pd1+ cells in each cluster. In the SPC;sgCul3 group, the percentage of Pd1+ CD4 and CD8 T cells were higher than those in the SPC;sgCul3 group (Fig. 4E, F, Table 2), suggesting increased immune escape that favoring tumorigenesis.

### Cul3 deficiency promotes PD1^high^ CD8 T cell accumulation

**Figure 5:**
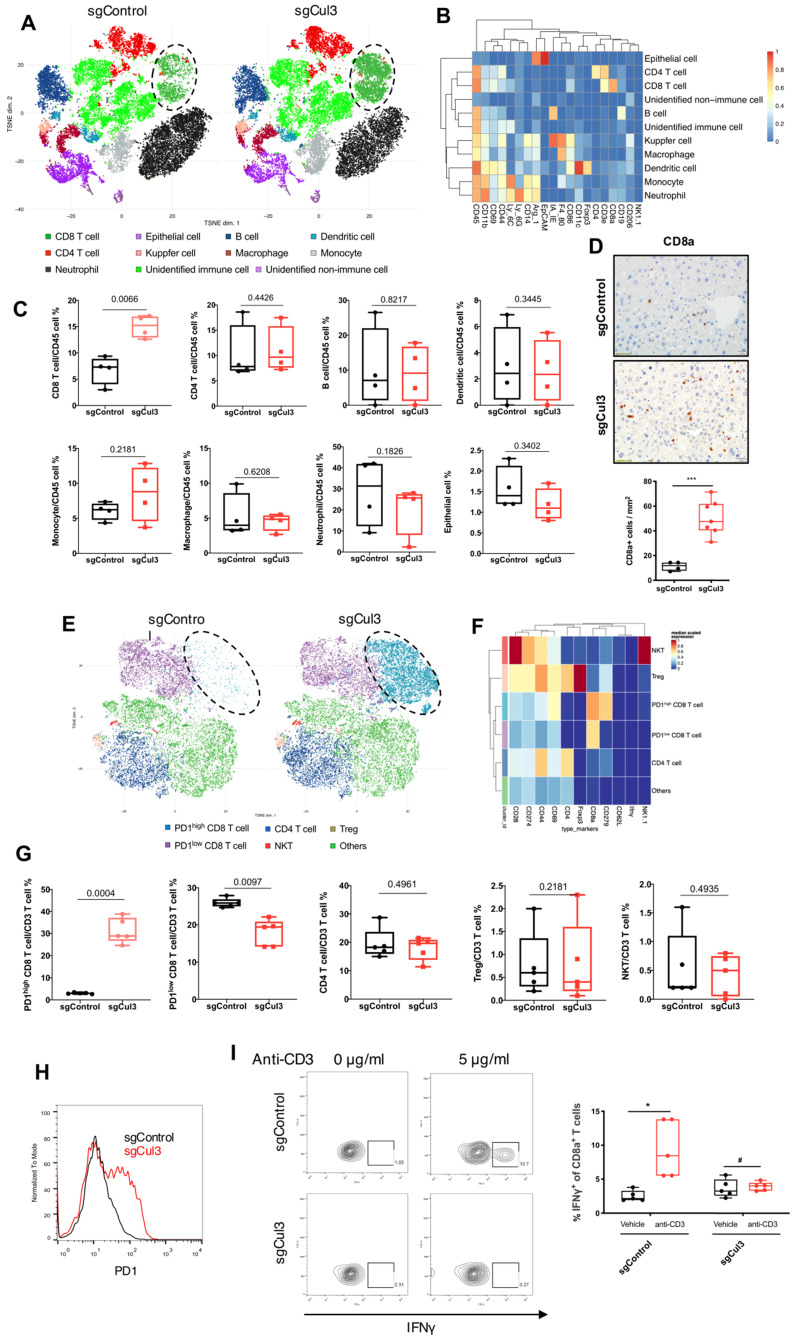
** Cul3 deficiency induces PD1^high^ CD8 T cell accumulation. (A)** CyTOF mapping of cells in livers at 2 weeks after injection with sgCul3 or sgControl (n=4). **(B)** The heat-map shows markers of each annotated lineage.** (C)** Quantification of each lineages in sgCul3 and sgControl groups (n=4). **(D)** Representative immunostaining of CD8a T cells in the livers, and quantification of CD8a cell numbers per area (n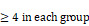). **(E)** CyTOF mapping of CD3 T cells in livers at 2 weeks after injection with sgCul3 or sgControl (n=5). **(F)** The heat-map shows markers of each annotated lineage. **(G)** Quantification of each lineages of CD3 T cells in sgCul3 and sgControl groups (n=5). **(H)** Flow cytometry analysis shows PD1 level on CD8 T cells in sgCul3 and sgControl groups. **(I)** Flow cytometry analysis of IFNγ production with anti-CD3 stimulation for 4 hours (n=5). *P<.05, #P>.05.

To further identify cell population landscapes supporting tumor initiation, we compared the cell composition in livers at 2 weeks after injection with sgCul3 or sgControl by CyTOF (cytometry by time of flight). tSNE analysis showed an increased frequency of CD8 T cell in the livers injected with sgCul3 compared with the frequency in the livers injected with sgControl, while there was no change in other cell lineages, including CD4 T cell, B cell, dendritic cell, monocyte, macrophage, neutrophil and epithelial cell (Fig. 5A-C). Consistent with the CyTOF data, it was also confirmed by immunohistochemistry staining of CD8a (Fig. 5D).

It is well known that CD8 T cells are the main cytotoxic cells against tumors. To further investigate the changes of T cell subtypes and the phenotype, we compared the CD3 T cells in livers at 2 weeks after injection with sgCul3 or sgControl by CyTOF. tSNE analysis showed an obvious increased frequency of PD1^high^ CD8 T cells in the livers injected with sgCul3 compared with the frequency in the livers injected with sgControl, while the PD1^low^ CD8 T cells decreased (Fig. 5E-G). Furthermore, flow cytometry analysis also revealed that compared with the control livers, the sgCul3-injected livers had much higher levels of PD1 on CD8 T cells (Fig. 5H). We next examined the regulation of Cul3 on CD8 T cells infiltration and PD1 expression in a human CC cohort, and it showed the negative correlation between Cul3 and CD8 T cells infiltration, as well as PD1 expression (Fig. S6).

As PD1 is regarded as an exhausted T cell marker, we then used IFNγ production assay to assess the potential cytotoxic activity of CD8 T cells. The T cells were isolated from the livers of sgCul3 or sgControl groups, and cultured in anti-CD28 antibody (2 µg/ml) containing medium, followed by stimulation with anti-CD3 antibody. Compared with the CD8 T cells from sgControl livers, the CD8 T cells from sgCul3 livers showed reduced capacity of IFNγ production with anti-CD3 antibody stimulation (Fig. 5I), suggesting the reduced potential activity for killing tumors.

### Cul3 deficiency promotes Areg secrection to shape tumor microenvironment

**Figure 6:**
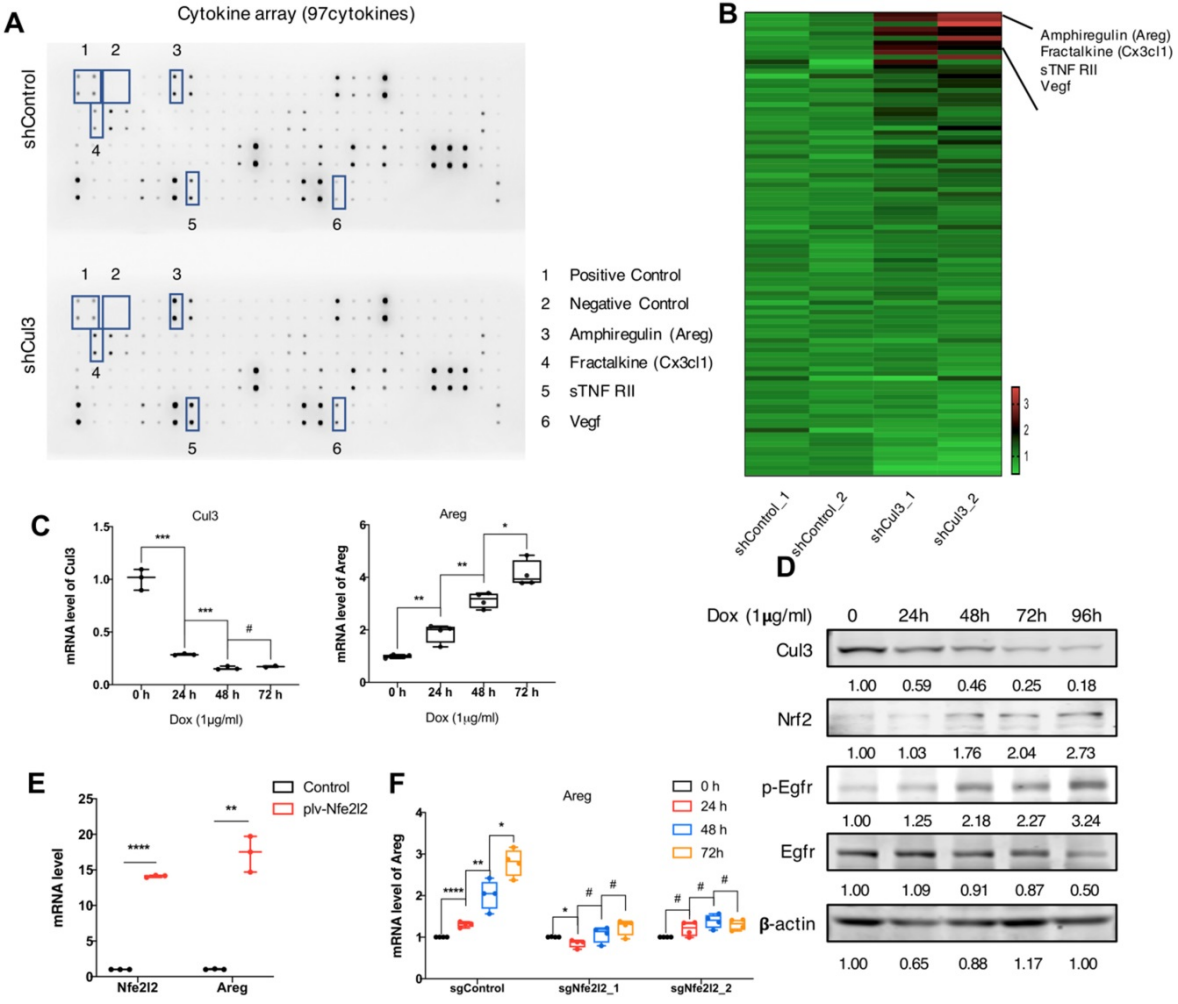
** Cul3 deficiency promotes Areg secrection to shape TME. (A)** Comparison of cytokines released by CC cells isolated from SPC mice. **(B)** Heat-map shows comparison of cytokine levels. **(C)** qRT-PCR shows mRNA levels of Areg. **(D)** Western blot shows the activated Egfr signalling and increased Nrf2 level. **(E)** qPCR shows increased Areg transcription by Nrf2 overexpression. **(F)** Cul3 deficiency induced Areg expression is blocked by knocking out Nfe2l2. Dox is the abbreviation of doxycycline, and all the Cul3-deficient cells are doxycyclin-inducible cell line.^ *^P<.05, ^**^P<.01, ^***^P<.001, ^****^P<.0001.

We next investigated if the Cul3 deficiency in cancer cells could elicit any effects on tumor microenvironment (TME). We compared cytokines that released to the supernantant by CC cells (273cc) isolated from SPC mice, and got 4 upregulated cytokines, including Amphiregulin (Areg), Fractalkine (Cx3cl1), sTNF RII and Vegf (Fig. 6A, B). Areg is a critical autocrine growth factor that interacts with the epidermal growth factor receptor (Egfr) and promotes epithelial cells growth. It was reported to be associated with pro-inflammatory cytokines production 33, and its high expression was detected in a variety of cancers 34. Here, we confirmed the increased transcription of Areg (Fig. 6C), as well as the increased Egfr phosphorylation in doxycycline (Dox)-induced Cul3-deficient CC cells (Fig. 6D). It was reported that Nrf2, the target of Cul3, could regulate Areg expression in esophageal epithelial cells 35. Here, we overexpressed the Nrf2 gene Nfe2l2, and detected the increased transcription of Areg in CC cells (Fig. 6E). To further demonstrate that Cul3 deficiency induced Areg expression was dependent on Nrf2, we knocked out the gene Nfe2l2, and found the reduced regulation of Areg by Cul3 (Fig. 6F). In summary, these results demonstrated that Cul3 inactivation induces the secretion of Areg through Nrf2, and promotes inflammatory cytokines production to shape the TME.

### Sorafenib enhances the anti-PD1 treatment efficiency in primary tumor

**Figure 7:**
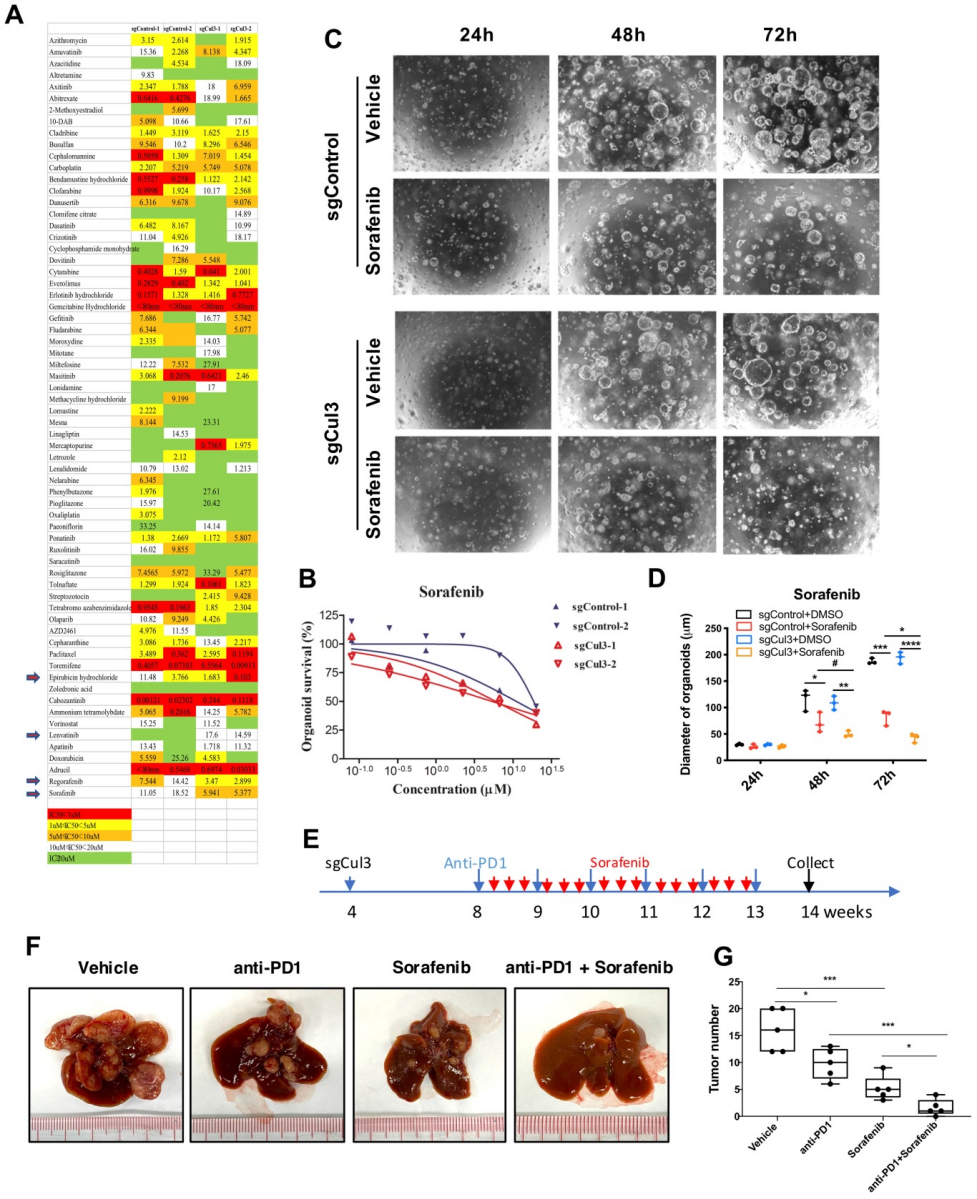
** Sorafenib enhances the anti-PD1 treatment efficiency in primary tumor. (A)** Comparison of drug sensitivity between wildtype and Cul3-deficient organoids with a drug library containing 132 drugs. **(B)** Confirmation of the enhanced response to sorafenib in Cul3-deficient organoids. **(C)** Representative figures showing repressed organoid growth after drug treatment. **(D)** Quantification of organoid diameter. **(E)** Schematic diagram of the *in vivo* treatment strategy. **(F)** Representative figures show the tumor burden in liver. **(G)** Quantification of tumor numbers (n=5). *P<.05, **P<.01, ***P<.001, ****P<.0001.

Because there is no effective therapeutic drug for CC, we next aimed to conduct drug screening with a drug library containing 132 drugs that were approved for cancer treatment (Supplementary Table 1) using 3-dimensional organoid technology. The screening results showed that 4 drugs became more effective in Cul3-deficient organoids than in wild-type organoids (Fig. 7A). Notably, sorafenib, the multikinase inhibitor for advanced HCC approved by the US Food and Drug Administration (FDA), showed enhanced sensitivity in Cul3-deficient organoids (Fig. 7B). To further confirm the enhanced sensitivity to sorafenib, we performed time-lapse observation of organoids under drug treatment. The data showed a faster decrease in organoid size in the sorafenib treatment groups (Fig. 7C, D).

Because CCs developed in SPC;sgCul3 mice were enriched exhausted PD1^high^ CD8 T cells, we decided to test the efficacy on inhibiting CC with the combination of the anti-PD1 antibody in primay tumor. We treated the mice with sgCul3 injection by oral with sorafenib every other day starting from age of 2 months, and anti-PD1 antibody by ip. injection every week starting from age of 2 month (Fig. 7E). Although either anti-PD1 antibody or sorafenib alone reduced tumor burden, the combination of them have a much higher efficiency, even elimilated the tumor (Fig. 7F, G). We believe that in the clinical setting, the availability of antibodies to reach cancer is quite limited; thus, our finding that the addition of sorafenib enhances the anti-PD1 treatment efficiency is of significance for further clinical testing.

## Discussion

Our data describe an efficient approach to conduct genome-scale screening of liver tumor suppressors *in vivo*. The previously used *in vivo* genetic screening methods, such as transposon or lentivirus insertional mutagenesis, usually analyzed a large number of mice, and candidate selection was labor intensive 36, 37. With the approach we utilized, we initially obtained about 200 candidate genes, whose disruption was associated with the accelerated CC formation. After using criteria for the appearance of at least 2 distinct sgRNAs for each gene, we identified 15 candidate genes and validated Cul3 as a suppressor of CC, while the candidacy of others remaining to be determined. Unlike the *in vitro* CRISPR knockout library screen 14, the *in vivo* screen is dependent on the tumor microenvironment, which is widely recognized to play a vital role in tumorigenesis 38.

Cul3 is the member of the largest E3 ubiquitin ligase family that functions on ubiquitination of a wide range of proteins that are involved in diverse cellular processes 39-41. A recent study of genome-wide screen *in vitro* showed that Cul3 deficiency induced an oncogenic transcriptional program, which lead to partial EMT and heightened genomic instability 42. However, its role in liver cancer is largely unexplored. A previous study indicated that KO Cul3 failed to induce liver cancer formation, although it induced stem cell expansion, and combined mutation of Cul3 and P53 in liver progenitor cells caused HCC 43. Our study confirmed that loss of function of Cul3 alone failed to induce liver cancer. However, its loss remarkably enhanced CC formation initiated by functional loss of Smad4 and Pten, both of which are frequently mutated in human CC 17, 19. These data suggest that Cul3 may serve as a helper of CC initiation and progression caused by deficiency of Smad4/Pten 7 and P53 43.

Our analysis revealed that the accelerated CC formaton in SPC mice was accompanied by increased expression of a number of Cul3 downstream genes, including Nrf2 and Cyclin D1 (Fig. 2F, G). Nrf2 is a target of the Cul3-Keap1 complex, which ubiquitinates and degrades it through the proteasome 26. Nrf2 activation has been revealed in a variety of cancers, such as liver, lung and pancreatic 27. Cyclin D1 is a target of the Cul3-Roc1 complex and is degraded by the ubiquitin-protease pathway 44. Its main function is to combine with Cdk4 or Cdk6 to control the G1/S transition, thus promoting cell proliferation 45. Cyclin D1 overexpression is prevalent in a variety of tumors, including lung, pancreatic, bladder and breast 46, 47. These data suggest that these increased oncogenic signaling, at least in part, contributed to the enhanced CC formation.

Nrf2 is a transcription factor that was able to mediate transcriptional induction of Areg, which plays a critical role in the inflammatory response 48, 49. In addition, we also found that Cul3 disruption elevated transcription of Stat1, leading to the increased levels of Stat1 protein and its phosphorylation, which is known to play a role in promoting inflammation 28, 29. Consistently, the upregulation of Stat1 and pStat1 was associated with the earliest time point of inflammation and also persisted along with the CC progression. Thus, we believe both the elevated levels of Nrf2 and pStat1 play a key in triggering CC initiation and progression.

Chronic inflammation helps early tumor lesion formation, which is less responsive to the type 1 immune response, thus allowing tumor initiation and progression 50. This process is mainly supported by myeloid-derived suppressor cells (MDSCs) and macrophages, and suppressed by cytotoxic T cells 51. In this study, there was obviously increased accumulation of stromal cells inside the SPC;sgCul3 tumors than inside the SPC tumors. Therefore, we examined the immune microenvironment and tumor microenvironment in the liver using immunohistochemistry and CyTOF analyses, and the data detected immune component changes as early as 2 weeks after the sgCul3 injection, especially for increased cytotoxic T cells. However, further analysis revealed features of reduced activity of these cells (Fig. 3 and 5).

The immune microenvironment is carefully orchestrated by chemotactic cytokines or chemokines, which induce immune cell trafficking, proliferation and activation. Several recent studies have reported the role of chemokines in immune cell infiltration in tumors 31, 32. Our data detected high levels of Ccl5-Ccr5 and Cxcl9/Cxcl10-Cxcr3 signaling in the early lesions one week after sgCul3 injection and maintained high levels thereafter. The remarkably increased levels of these cytokines induced by Cul3 deficiency could attract CD8 T cells to tumor cells and explained their accumulation in the liver. Moreover, these cytokines might also passage exhaustion signaling to T cells. In addition, the exhaustion of cytotoxic T cells could also be caused by inflammation 52, 53. Thus, either these cytokines or inflammatory signals, or the combination of both, could be responsible for cytotoxic T cell exhaustion, allowing premalignant cholangiocytes to escape from elimination and develop into a tumor.

Another significant finding is that we detected increased expression level of PD1 on CD8 T cells, which may also contribute significantly to the accumulation of exhausted cytotoxic T cells. PD-L1 is commonly overexpressed on tumor cells or nontransformed cells in the tumor microenvironment 54. PD-L1 expressed on tumor cells binds to PD1 receptors on activated T cells, which leads to cytotoxic T cell inhibition, allowing the growth of cancer cells 54. Recently, US-FDA and China National Medical Products Administration (NMPA) have approved the use of PD1 blockade on advanced HCC patients, who are already treated with sorafenib or oxaliplatin. Our data indicated the high efficiency of anti-PD1 antibody for CC treatment, and the combination of sorafenib with anti-PD1 antibody significantly further increased the efficacy. Of note, sorafenib is an approved drug for HCC treatment, and its high efficacy on CC illustrated here is determined by our screening with 132 anticancer drugs. Thus, our data not only reflect the similarity between HCC and CC, but also suggest the necessicity of clinical trial for such combinatory treatments of human CC.

## Conclusions

This study introduces an efficient approach for genome-scale screening of liver tumor suppressors *in vivo*, and identifies Cul3 as a suppressor of CC. Cul3 deficiency promotes expression of oncogenes, including Nrf2, Cyclin D1, and Areg-Egfr signaling, that initiate CC formation and progression. Meanwhile, Cul3 deficiency increases the production of Areg, which may induce inflammation in the liver, and promote exhausted PD1^high^ CD8 T cells accumulation, and accelerate cholangiocyte cell expansion at an early stage. The scRNA-Seq reveales reduced activity of cytotoxic T cells in tumors from SPC;sgCul3 mice compared with that in SPC mice. These findings prompt us to test the anti-PD1/PD-L1 blockade, and the data shows an effective response, and the effect is enhanced by the combination with sorafenib in primary tumor. These results enhance our understanding of the molecular mechanisms of liver cancer development and pave the way for clinical therapy.

## Supplementary Material

Supplementary figures and table.

## Figures and Tables

**Table 1:** The sgRNAs corresponding to top candidate genes

Genes	UID of sgRNAs enriched in top 200 enrichment list
Trp53	MGLibA_56035, MGLibA_56033, MGLibA_56034
Cul3	MGLibA_12475, MGLibA_12477, MGLibA_12476
Fbxw7	MGLibA_18106, MGLibA_18108, MGLibA_18107
Inppl1	MGLibA_26092, MGLibA_26094, MGLibA_26093
Bgn	MGLibA_06851, MGLibA_06850, MGLibA_06849
Dhcr7	MGLibA_14046, MGLibA_14045
Unc80	MGLibA_57346, MGLibA_57345
Apc	MGLibA_04599, MGLibA_04600
Spta1	MGLibA_51191, MGLibA_51192
Trp53bp1	MGLibA_56011, MGLibA_56009
Rad23b	MGLibA_44346, MGLibA_44345
Mlh1	MGLibA_31447, MGLibA_31449
Nrxn3	MGLibA_34816, MGLibA_34817
Slc6a13	MGLibA_49644, MGLibA_49643
Zbtb44	MGLibA_60094, MGLibA_60095

The CRISPR knockout library (#1000000052, Addgene) contains 3 sgRNAs for each gene and genes that were disrupted by at least two different sgRNAs were listed.

**Table 2:** Cell proporation of Pd1- or Pd1+ T cells in SPC and SPC;sgCul3 mice

Group	SPC;sgControl (1221 cells)						SPC;sgCul3 (1992 cells)					
Cell type	CD4	CD8	Cycling	Cxcr6+T cells	NK	Treg	CD4	CD8	Cycling	Cxcr6+T cells	NK	Treg
Proporation % ±SD (cell number)	34.08±4.94 (431)	20.94±4.92 (276)	4.62±2.25 (63)	11.89±4.16 (156)	2.53±0.59 (32)	21.07±4.70 (263)	25.88±8.68 (612)	34.28±11.19 (762)	4.87±1.62 (92)	5.13±1.42 (109)	2.09±0.47 (41)	21.41±8.38 (376)
PD1- % ±SD (cell number)	97.45±1.42 (420)	95.71±2.72 (263)	66.57±11.59 (45)	70.45±12.42 (108)	100.00±0.00 (32)	76.34±10.91 (203)	91.87±3.42 (576)	81.17±9.75 (600)	55.57±19.57 (51)	63.01±6.28 (71)	96.11±4.84 (39)	66.91±7.84 (248)
PD1+ % ±SD (cell number)	2.55±1.42 (11)	4.29±2.72 (13)	33.43±11.59 (18)	29.55±12.42 (48)	0.00±0.00 (0)	23.66±10.91 (60)	8.13±3.42 (36)	18.83±9.75 (162)	44.43±19.57 (41)	36.99±6.28 (38)	3.89±4.84 (2)	33.09±7.84 (128)

## Abbreviations

ALT: Alanine transaminase
Areg: Amphiregulin
AST: Aspartate Aminotransferase
CC: cholangiocarcinoma
Cul3: Cullin3
CyTOF: cytometry by time of flight
GeCKO library: genome-scale CRISPR knockout library
HCC: hepatocellular carcinoma
LDH: lactate dehydrogenase
NGS: next generation sequencing
sgRNA: single-guide RNA
SP: *Smad4^co/co^;Pten^co/co^*
SPC: *Smad4^co/co^;Pten^co/co^;Alb-Cre*
