# Supplementary material for: Cullin3 deficiency shapes tumor microenvironment and promotes cholangiocarcinoma in liver-specific Smad4/Pten mutant mice
Source: Int J Biol Sci. 2021 Oct 11;17(15):4176–91. doi: 10.7150/ijbs.67379 (PMC8579464; doi:10.7150/ijbs.67379)
Supplement: Supplementary file 1 — Supplementary figures and table. [file ijbsv17p4176s1.pdf]

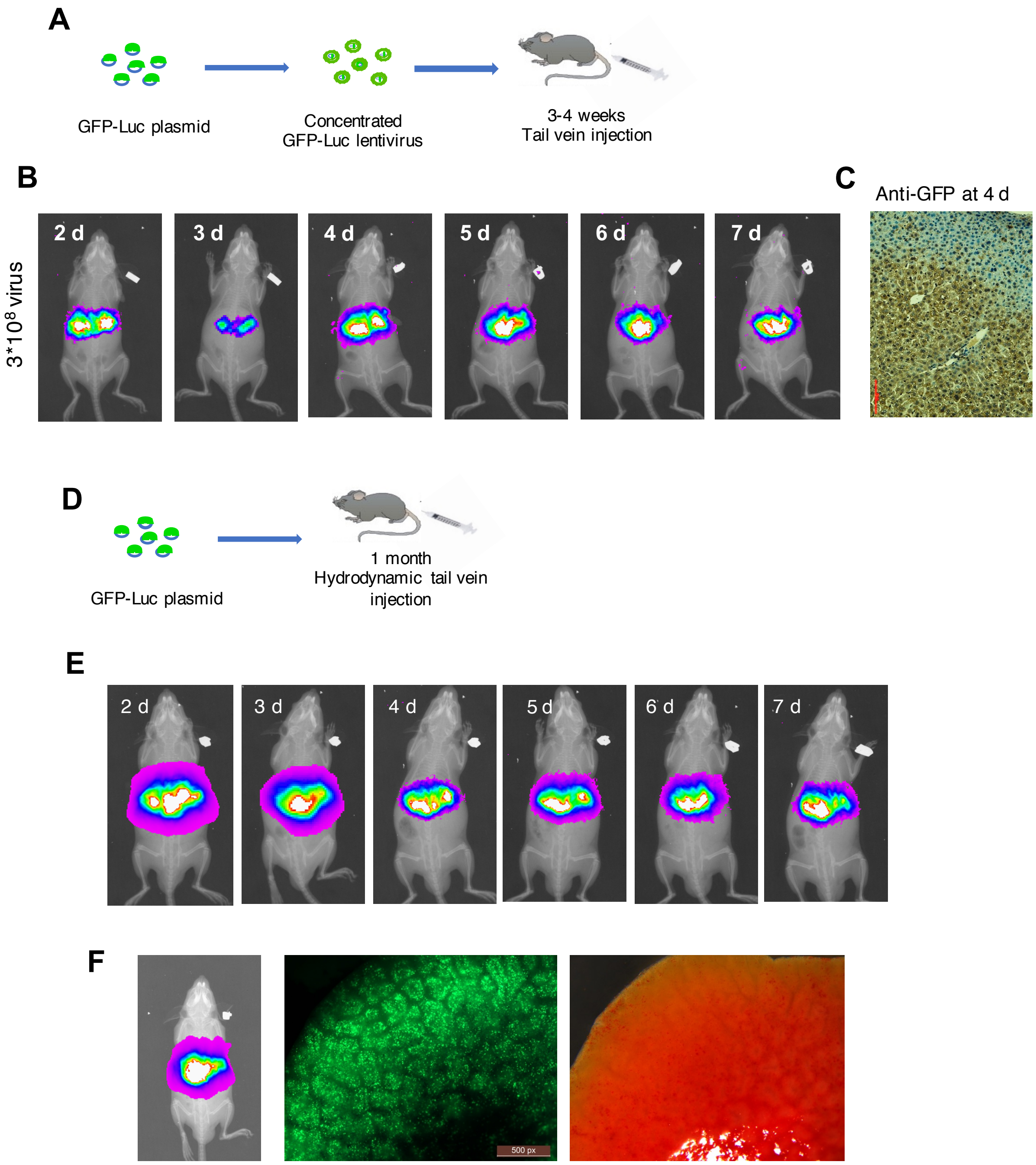


**Figure S1. Methods of gene delivery to liver. (A)** Outline of tail vein injection of lentivirus mediated GFP-Luc reporter. **(B)** Representative figures show luciferase signal during the following week after lentivirus injection. **(C)** Paraffin sections of liver are stained with antibody against GFP. **(D)** Outline of hydrodynamic tail vein injection of GFP-Luc plasmid. **(E)** The consecutive figures show dynamic luciferase signals during the following week after injection of GFP-Luc plasmid. **(F)** Representative images show strong and uniform GFP signal in liver 4 days after injection.

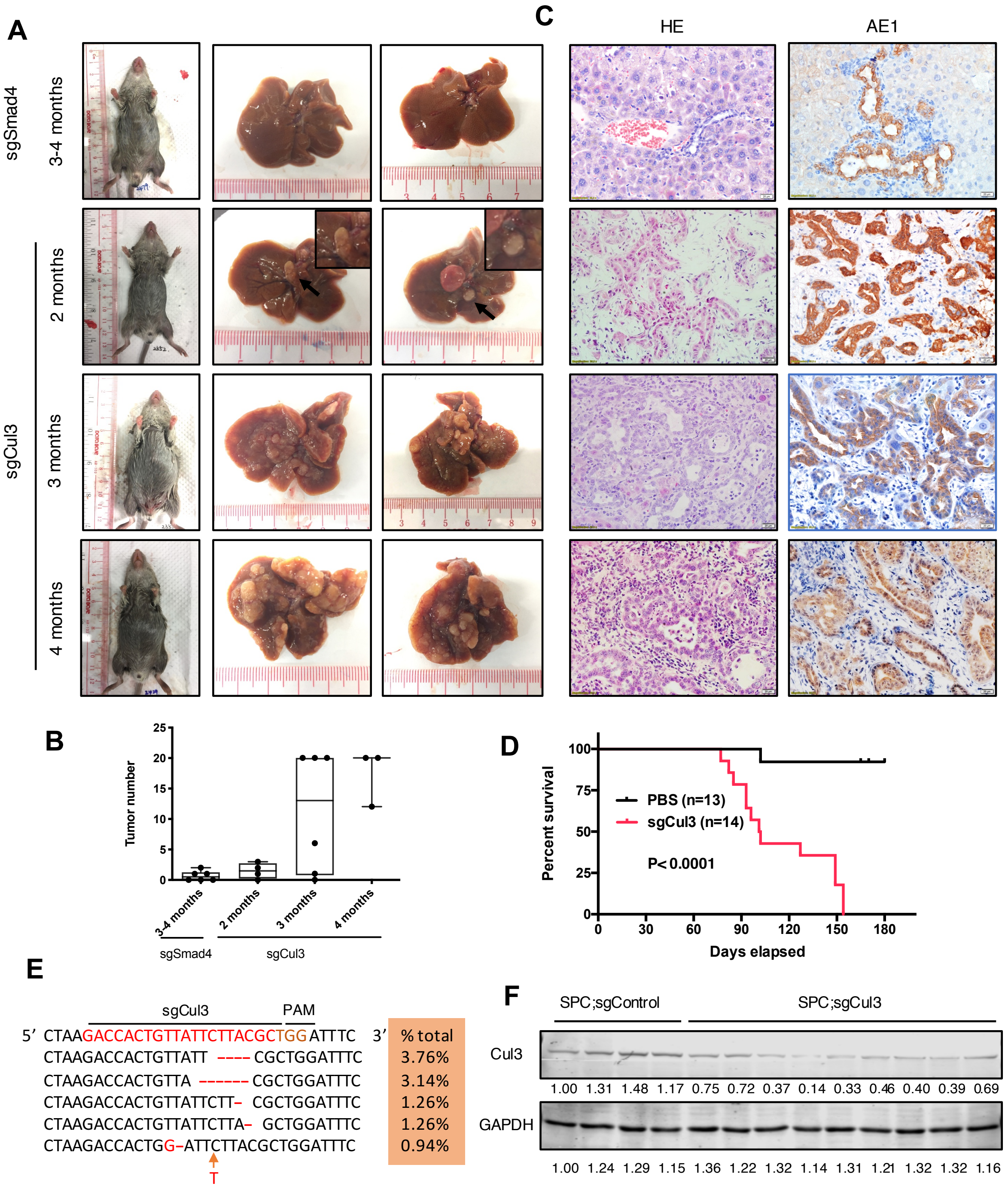


**Figure S2. Inactivation of Cul3 induces CC formation in SPC mice. (A)** Representative images of the liver at 2, 3 and 4 months of age, which are hydrodynamically injected with sgRNA targeting Cul3 at the age of 1 month. **(B)** Quantification of tumor numbers at 2, 3 and 4 months of age. **(C)** Histological analysis of tumors. **(D)** Kaplan-Meier survival curve for SPC and SPC;sgCul3 mice ($P < .0001$). **(E)** High-throughput sequencing shows indel mutations of the Cul3 targeting site and the percentage of each read. **(F)** Western blot analysis shows lower Cul3 protein levels in tumors from SPC;sgCul3 mice at 3-4 months compared with tumors from SPC;sgControl mice at 6-7 months.

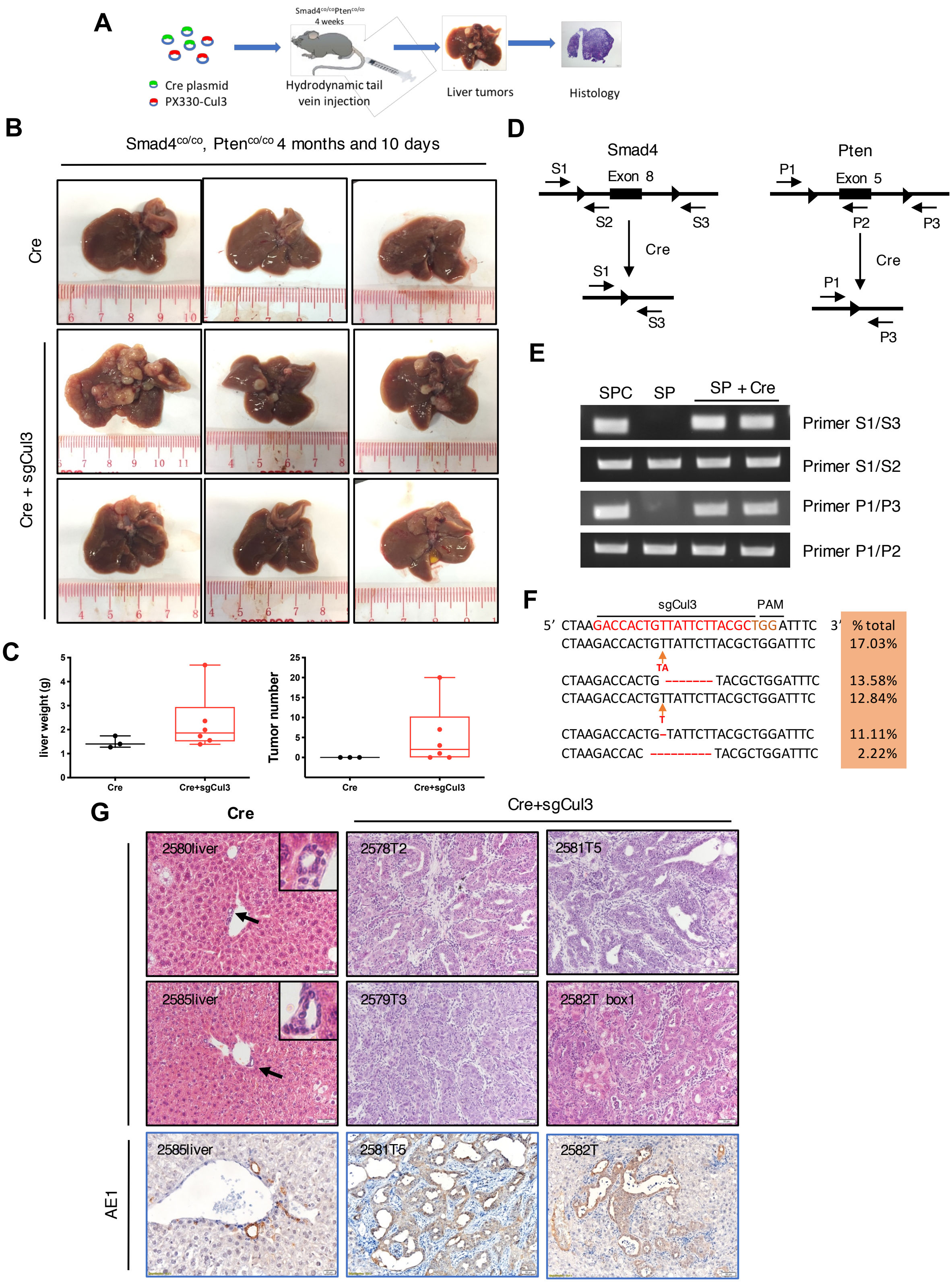


**Figure S3. Triple knockout of Smad4, Pten and Cul3 induces liver tumors in wildtype mice. (A)** Outline of hydrodynamic tail vein injection of Cre plasmid and sgRNA targeting Cul3. **(B)** Macroscopic images of livers injected with Cre or Cre and sgCul3. **(C)** Statistics of tumor number and liver weight. **(D)** Schematic diagram shows the exon deletion by Cre. **(E)**

PCR products amplified by indicated primers demonstrate the exon deletion. The Alb-Cre in first column is set as a positive control, while the senond column without Cre plasmid injection is set as a negative control. **(F)** NGS sequence shows indel mutations of Cul3 targeting site and the percentage of each read. **(G)** Histology analysis of tumors. AE1 indicates cholangiocyte.

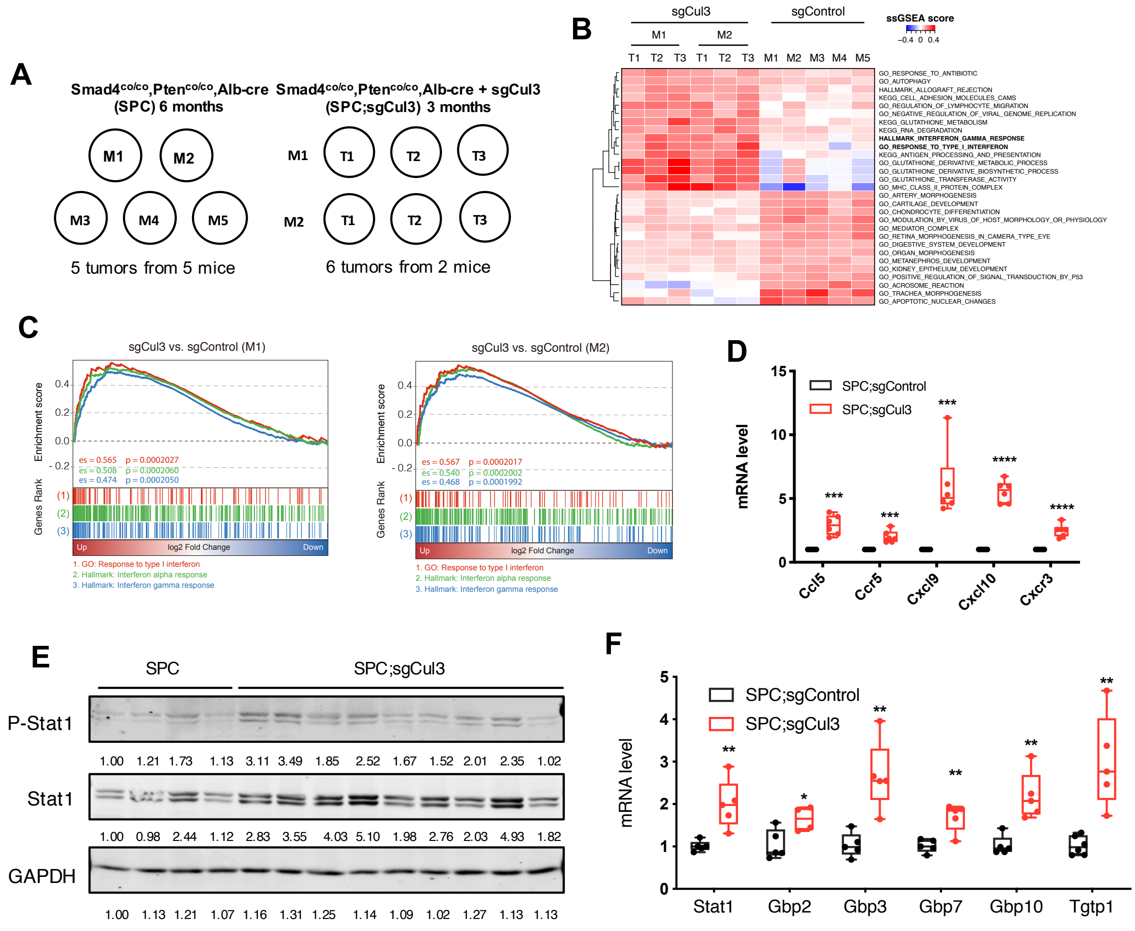

**Figure S4. Cul3 deficiency induces inflammation in the tumor. (A)** Diagram showing the scheme used for transcriptome analyses. **(B)** The heat-map shows the up- or down-regulated pathways analysed by Gene set enrichment analysis (GSEA). M1-M5 in SPC group indicate 5 mice, and T1-T3 in SPC;sgCul3 group indicate 3 tumors from each mouse. **(C)** GSEA profiles show enrichment of gene sets associated with inflammation. **(D)** RNA-seq data shows ligand-receptor expression changes between tumors from SPC;sgCul3 and SPC mice. **(E)** Western blot and **(F)** qRT-PCR results reveal the activation of Stat1 pathway. $^{*}P<.05$, $^{**}P<.01$, $^{***}P<.001$, $^{****}P<.0001$, $^{\#}P>.05$.

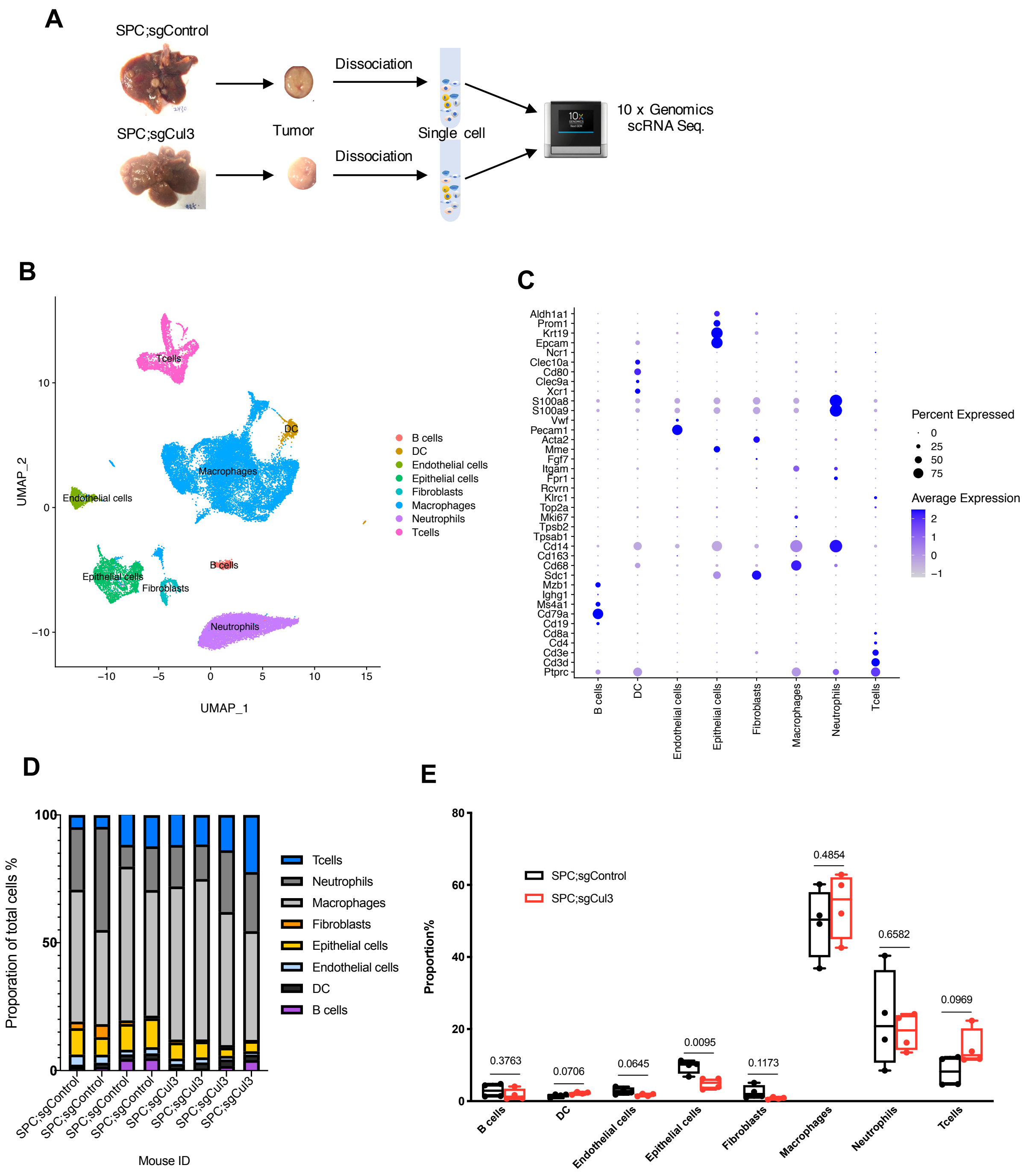


**Figure S5. scRNA-Seq identifies major cell clusters in tumors from SPC and SPC;sgCul3 mice. (A)** Schematic diagram shows sample collection and single cell preparation for 10× Genomics. The tumors with approximately equal size are isolated from 3-month old SPC;sgCul3 mice and 6-month-old SPC mice. **(B)** t-SNE plots show the identified cell clusters. **(C)** Heat-map shows the expression levels of marker genes for each cell population. **(D, E)** Quantification of each lineages.

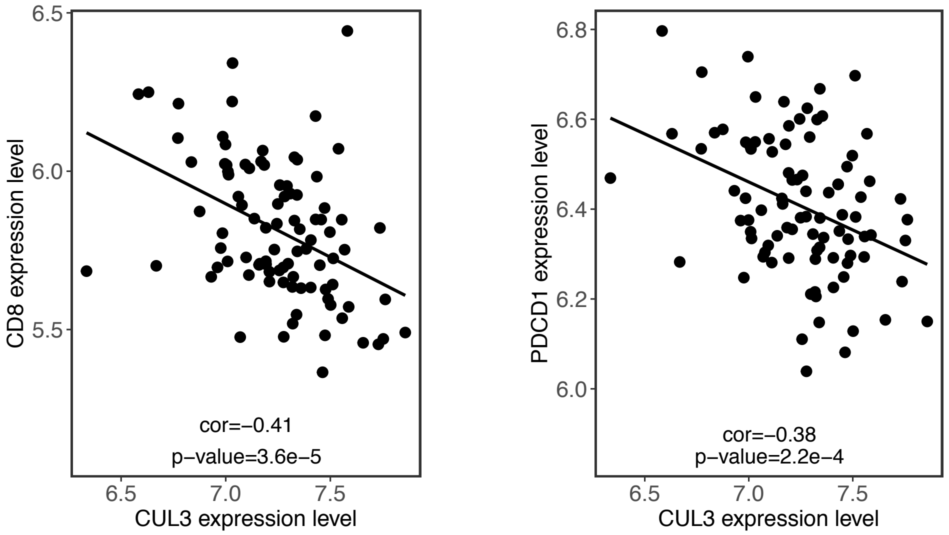


**Figure S6. Correlation between CUL3 and CD8 or PD1 in human CC cohort (GSE76297).**

**Supplementary Table 1 The drug library containing 132 drugs.**

| No. | Drug | Indication | Target | Approved status |
|---|---|---|---|---|
| 1 | 10-DAB | Cancer chemotherapy | Microtubule | Not approved (precursor of the approved taxane) |
| 2 | 2-Methoxyestradiol | Cancer targeted therapy | HIF | Not Approved (Phase 2 Clinical trials) |
| 3 | Abiraterone acetate | Cancer hormone therapy | Androgen synthesis | FDA |
| 4 | Abitrexate | Cancer chemotherapy | DHFR | FDA |
| 5 | Adrucil | Cancer chemotherapy | DNA/RNA Synthesis | FDA |
| 6 | Altretamine | Cancer chemotherapy | DNA/RNA Synthesis | FDA (Olaparib approved) |
| 7 | Amuvatinib | Cancer targeted therapy | c-Kit, PDGFR, FLT3 | INN, USAN (Phase 2 clinical trials) |
| 8 | Anastrozole | Cancer hormone therapy | Aromatase | FDA |
| 9 | Ancitabine hydrochloride | Cancer chemotherapy | DNA/RNA Synthesis | INN, MI |
| 10 | Artemether | | | |
| 11 | Aspirin | | | |
| 12 | Atazanavir sulfate | | | |
| 13 | Axitinib | Cancer targeted therapy | VEGFR, PDGFR, c-Kit | FDA |
| 14 | Azacitidine | Cancer chemotherapy | DNA/RNA Synthesis | FDA |
| 15 | Azaguanine-8 | Cancer chemotherapy | DNA/RNA Synthesis | Not Approved (Clinical trials) |
| 16 | Azithromycin | | | |
| 17 | Bendamustine hydrochloride | Cancer chemotherapy | DNA/RNA Synthesis | FDA |
| 18 | Bepotastine Besilate | | | |
| 19 | Bergapten | | | |
| 20 | Bindarit | Anti-inflammatory | NF-kB | |
| 21 | Bleomycin sulfate | Cancer chemotherapy | DNA/RNA Synthesis | FDA |
| 22 | Bosutinib | Cancer targeted therapy | Src,Abl | FDA |
| 23 | Busulfan | Cancer chemotherapy | DNA/RNA Synthesis | FDA |
| 24 | Cantharidin | | | |
| 25 | Capecitabine | Cancer chemotherapy | DNA/RNA Synthesis | FDA |
| 26 | Carbazochrome sodium sulfonate | | | |
| 27 | Carboplatin | Cancer chemotherapy | DNA/RNA Synthesis | FDA |
| 28 | Carmofur | Cancer chemotherapy | DNA/RNA Synthesis | FDA |
| 29 | Cephalomannine | | | |
| 30 | Chlorambucil | Cancer chemotherapy | DNA/RNA Synthesis | FDA |
| 31 | Cinacalcet hydrochloride | | | |
| 32 | Cisplatin | Cancer chemotherapy | DNA/RNA Synthesis | FDA |
| 33 | Cladribine | Cancer chemotherapy | DNA/RNA Synthesis | FDA |
| 34 | Clofarabine | Cancer chemotherapy | DNA/RNA Synthesis | FDA |
| 35 | Clomifene citrate | | | |
| 36 | Clorsulon | | | |
| 37 | Crizotinib | Cancer targeted therapy | c-Met | FDA |
| 38 | Cyclophosphamide monohydrate | Cancer chemotherapy | DNA/RNA Synthesis | FDA |
| 39 | Cytarabine | Cancer chemotherapy | DNA/RNA Synthesis | FDA |
| 40 | Dacomitinib | Cancer targeted therapy | EGFR | INN, USAN (Phase 2 clinical trials) |

| | | | | |
|---|---|---|---|---|
| 41 | Danusertib | Cancer targeted therapy | Aurora kinase | INN (Phase 2 clinical trials) |
| 42 | DAPT (GSI-IX) | | | |
| 43 | Dasatinib | Cancer targeted therapy | Src, Bcr-Abl, c-Kit | FDA |
| 44 | Diethylstilbestrol | | | |
| 45 | Dovitinib | Cancer targeted therapy | FGFR | INN, USAN (Phase 2 clinical trials) |
| 46 | Doxifluridine | Cancer chemotherapy | DNA/RNA Synthesis | INN, JAN |
| 47 | Doxorubicin | Cancer chemotherapy | Topoisomerase | FDA |
| 48 | Epirubicin hydrochloride | Cancer chemotherapy | Topoisomerase | FDA |
| 49 | Erlotinib hydrochloride | Cancer targeted therapy | EGFR | FDA |
| 50 | Esomeprazole sodium | | | |
| 51 | Estramustine | Cancer chemotherapy | Microtubule | FDA |
| 52 | Everolimus | Cancer targeted therapy | mTOR | FDA |
| 53 | Fludarabine | Cancer chemotherapy | DNA/RNA Synthesis | FDA |
| 54 | Flunarizine dihydrochloride | | | |
| 55 | Flutamide | Cancer hormone therapy | Androgen receptor | FDA |
| 56 | Formestane | Cancer hormone therapy | Aromatase | INN, BAN |
| 57 | Ftorafur | Cancer chemotherapy | DNA/RNA Synthesis | FDA |
| 58 | Fulvestrant | Cancer hormone therapy | Estrogen/progestogen Receptor | FDA |
| 59 | Gefitinib | Cancer targeted therapy | EGFR | FDA |
| 60 | Gemcitabine Hydrochloride | Cancer chemotherapy | DNA/RNA Synthesis | FDA |
| 61 | Geniposidic acid | | | |
| 62 | Genistein | Cancer chemotherapy | Topoisomerase | Not Approved (Phase 2 Clinical trials) |
| 63 | Histamine dihydrochloride | Cancer immunotherapy | Enhancing IL-2 | FDA |
| 64 | Ifosfamide | Cancer chemotherapy | DNA/RNA Synthesis | FDA |
| 65 | Imatinib mesylate | Cancer targeted therapy | PDGFR, c-Kit, Bcr-Abl | FDA |
| 66 | Irinotecan | Cancer chemotherapy | Topoisomerase | FDA |
| 67 | Itraconazole | | | |
| 68 | Lamotrigine | | | |
| 69 | Lapatinib Ditosylate | Cancer targeted therapy | EGFR, HER2 | FDA |
| 70 | Lenalidomide | Cancer immunotherapy | Immunomodulator | FDA |
| 71 | Letrozole | Cancer hormone therapy | Aromatase | FDA |
| 72 | Linagliptin | | | |
| 73 | Lomustine | Cancer chemotherapy | DNA/RNA Synthesis | FDA |
| 74 | Lonidamine | Cancer targeted therapy | Hexokinase | INN, BAN, (Phase 3 clinical trials) |
| 75 | Masitinib | Cancer targeted therapy | c-Kit, PDGFR, FGFR | INN (Phase 2/3 clinical trials) |
| 76 | MDV3100 | Cancer hormone therapy | Androgen Receptor | FDA |
| 77 | Mechlorethamine HCL | | | |
| 78 | Megestrol acetate | Cancer hormone therapy | Progesterone analog | INN, BAN |
| 79 | Mercaptopurine | Cancer chemotherapy | DNA/RNA Synthesis | FDA |
| 80 | Mesna (Uromitexan, Mesnex) | | | |
| 81 | Methacycline hydrochloride | | | |
| 82 | Methazolastone | Cancer chemotherapy | DNA/RNA Synthesis | FDA |
| 83 | Miltefosine | | | |

| | | | | |
|---|---|---|---|---|
| 84 | Mitotane | Cancer chemotherapy | Adrenal cortex | FDA |
| 85 | Moroxydine | | | |
| 86 | Naloxone HCl | | | |
| 87 | Nelarabine | Cancer chemotherapy | DNA/RNA Synthesis | FDA |
| 88 | Nilotinib | Cancer targeted therapy | Bcr-Abl | FDA |
| 89 | Nilvadipine | Cancer targeted therapy | PARP | FDA |
| 90 | Oxaliplatin | Cancer chemotherapy | DNA/RNA Synthesis | FDA |
| 91 | Paclitaxel | Cancer chemotherapy | Microtubule | FDA |
| 92 | Paeoniflorin | Anti-inflammatory | NF-kB | |
| 93 | Pazopanib hydrochloride | Cancer targeted therapy | VEGFR, PDGFR, c-Kit | FDA |
| 94 | Pemetrexed disodium | Cancer chemotherapy | DHFR | FDA |
| 95 | Phenylbutazone | | | |
| 96 | Phenylbutyric acid | | | |
| 97 | Pioglitazone | | | |
| 98 | Pomalidomide | Cancer immunotherapy | Immunomodulator | FDA |
| 99 | Ponatinib | Cancer targeted therapy | Abl | FDA |
| 100 | Procarbazine hydrochloride | Cancer chemotherapy | DNA/RNA Synthesis | FDA |
| 101 | Regorafenib | Cancer targeted therapy | VEGFR | FDA |
| 102 | Rosiglitazone | | | |
| 103 | Ruxolitinib | Cancer targeted therapy | JAK | FDA |
| 104 | Saracatinib | Cancer targeted therapy | Src, Bcr-Abl | INN, USAN (Phase 2 clinical trials) |
| 105 | Sorafenib | Cancer targeted therapy | VEGFR, PDGFR, Raf | FDA |
| 106 | Streptozotocin | Cancer chemotherapy | DNA/RNA Synthesis | FDA |
| 107 | Sulindac | | | |
| 108 | Sunitinib malate | Cancer targeted therapy | VEGFR, PDGFR, c-Kit, Flt | FDA |
| 109 | Temocapril hydrochloride | | | |
| 110 | Temsirolimus | Cancer targeted therapy | mTOR | FDA |
| 111 | Teniposide | Cancer chemotherapy | Topoisomerase | FDA |
| 112 | Tofacitinib citrate | Cancer targeted therapy | JAK | FDA |
| 113 | Tolbutamide | | | |
| 114 | Tolnaftate | | | |
| 115 | Topotecan hydrochloride | | | |
| 116 | Tretinoin | Cancer targeted therapy | PML-RAR fusion protein | FDA |
| 117 | Vandetanib | Cancer targeted therapy | VEGFR | FDA |
| 118 | Vatalanib | Cancer targeted therapy | VEGFR, PDGFR, c-Kit | INN (Phase 2 clinical trials) |
| 119 | Vemurafenib | Cancer targeted therapy | B-Raf | FDA |
| 120 | Vinorelbine | Cancer chemotherapy | Microtubule | FDA |
| 121 | Vismodegib | Cancer targeted therapy | Hedgehog | FDA |
| 122 | Vorinostat | Cancer targeted therapy | HDAC | FDA |
| 123 | XL-184 (Cabozantinib) | Cancer targeted therapy | c-Met | |
| 124 | Zoledronic acid | | | |
| 125 | Toremifene | Cancer hormone therapy | Estrogen receptor | |
| 126 | Cepharanthine | Anti-inflammatory | | |
| 127 | AZD2461 | Cancer targeted therapy | PARP | Not Approved (Phase 1 clinical trials) |

| | | | | |
|---|---|---|---|---|
| 128 | Olaparib | Cancer targeted therapy | PARP | FDA |
| 129 | Tetrabromo azabenzimidazole | Cancer targeted therapy | Casein kinase-2 (CK2) | |
| 130 | Ammonium tetramolybdate | Cancer chemotherapy | Chelating copper | Not Approved (Phase 2 clinical trials) |
| 131 | Apatinib | Cancer targeted therapy | VEGFR, PDGFR, c-Kit | FDA |
| 132 | Lenvatinib | Cancer targeted therapy | VEGFR, PDGFR, Raf | FDA |
